# RNA sequencing analysis of the human retina and associated ocular tissues

**DOI:** 10.1038/s41597-020-0541-4

**Published:** 2020-06-24

**Authors:** Scott T. Schumacker, Krista R. Coppage, Ray A. Enke

**Affiliations:** 1 Department of Biology, James Madison University, Harrisonburg, VA 22807 USA; 2 Center for Genome & Metagenome Studies, James Madison University, Harrisonburg, VA 22807 USA

**Keywords:** RNA sequencing, Retina, Transcriptomics

## Abstract

The retina is a stratified layer of sensory neurons lining the posterior portion of the eye. In humans, fine detail and color vision are enabled by the macula, a central region of the retina dense in cone photoreceptors (PRs). Achromatic low light and peripheral vision are facilitated by rod PRs found with increasing density outside the macula in the peripheral retina. The outer retina is nourished by choroidal blood flow regulated by a single layer of intervening retinal pigment epithelial (RPE) cells. Existing human retinal transcriptome projects have been critical for studying aspects of retinal development and disease however, there are currently no publicly available data sets accurately describing the aging human central retina, peripheral retina, and supporting RPE/choroid. Here we used Illumina RNA sequencing (RNA-seq) analysis to characterize the mRNA transcriptome of rod and cone PR-enriched human retina as well as supporting macular RPE/choroid tissue. These data will be valuable to the vision research community for characterizing global changes in gene expression in clinically relevant ocular tissues.

## Abstract (summary)

**Table:** 

Measurement(s)	RNA • transcriptome
Technology Type(s)	RNA sequencing
Factor Type(s)	type of ocular tissue
Sample Characteristic - Organism	Homo sapiens

Machine-accessible metadata file describing the reported data: 10.6084/m9.figshare.12464540

## Background & Summary

Since the emergence of commercial Next Generation Sequencing (NGS) technology ~13 years ago, genomics data represents one of the most proliferative Big Data domains with unprecedented growth projected by the year 2025^1^. Once made publicly available, genome-wide experiments provide the research community with valuable data that can be subsequently mined to further scientific knowledge. Beyond submission to public repositories, detailed curation of these datasets is critical for accurate interpretation and repurposing of NGS data. NGS technology has allowed for extensive eukaryotic transcriptome analysis using a wide range of tissues and single cells harvested from a variety of species^2,3^. These RNA-seq analyses have become the gold standard for in depth characterization of global differences in transcript expression as well as the accumulation of novel tissue and cell-specific transcript isoforms.

The vision research community has greatly benefited over the past decade from a deluge of RNA-seq and associated NGS data describing the neural retina and its supporting tissues^4,5^. Non-mammalian models such as zebrafish and chickens have been successfully employed to characterize global changes in chromatin organization^6–8^ and associated transcriptional networks^9–11^ during vertebrate retinal development. Mammalian models, particularly the mouse, have been more extensively used to integrate retinal transcriptome and chromatin organization NGS data sets^12–15^. Collectively, these data have been critical for the determination of chromatin states required for cell-type specific transcription in vertebrate retinal neurons as well as for modeling rare retinal degenerative diseases. Ultimately though, detailed aspects of human retinal development as well as genetically complex human retinal diseases such as age-related macular degeneration (AMD) have proven difficult to model in non-human animals.

The experiment described here is part of a larger ongoing project within the James Madison University’s Center for Genome & Metagenome Studies (CGEMS) investigating transcriptional regulation in the developing, mature, aging, and diseased vertebrate retina. Within the human retina, fine detail and color vision required for reading, facial recognition, and many other day-to-day activities are enabled by the macula, a 5.5 mm central region dense in cone photoreceptors (PRs). Achromatic low light and peripheral vision are facilitated by rod PRs found with sharply increasing density outside of the macula moving toward the peripheral regions of the retina^16^. Rod and cone PR neurons orchestrate cell-specific transcriptional networks critical for differentiation and proper function^17^. Farkis and colleagues completed the first comprehensive RNA-seq analysis of whole retina tissue collected from three adult human donors^18^. This analysis provided initial insight into the complexity of the whole human retinal transcriptome including novel exons and novel transcripts expressed in whole retinal tissue. A subsequent study sampling central and peripheral regions of eight adult human retinas hinted at transcriptional networks controlling rod and cone-specific PR function^19^. However, a 8 mm region of the central retina was collected in this study extending to an area 1.5X beyond the anatomical macular circumference and thereby resulting in rods representing the majority PR cell type in both sample groups. Additionally, Li and colleagues report limited read quality and sampling metrics in their study.

**Fig. 1:**
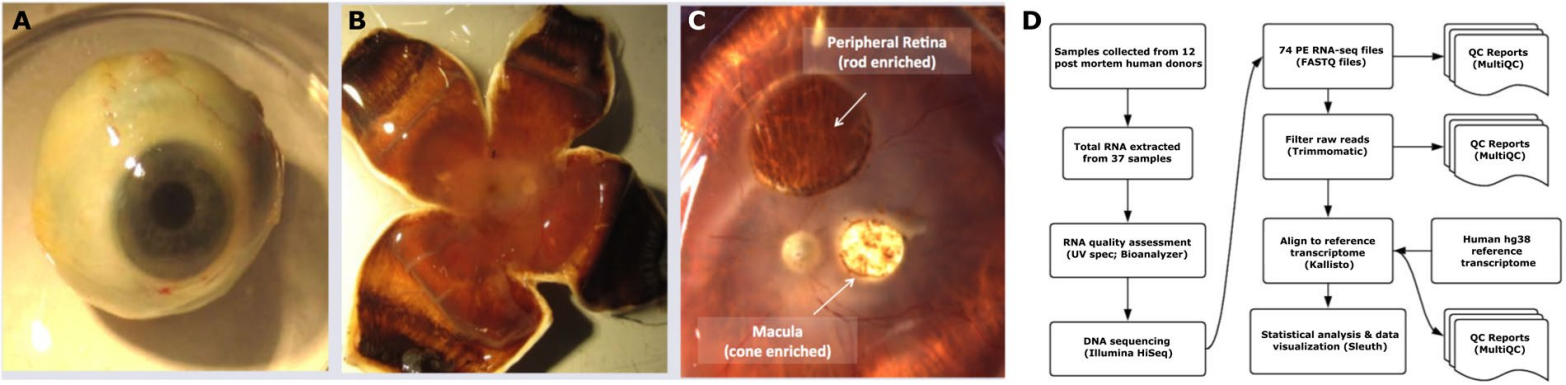
Overview of human donor eye dissection, sample collection and downstream analysis. Pairs of whole globe human donor eyes were procured and dissected so that whole corneas were collected (**A**) prior to making radial cuts down the side of the globe to expose the retina (**B**). The central retina and adjacent RPE/choroid layers were collected using a 3 mm biopsy punch in the center of each retina while the rod-rich peripheral retina was collected using a 6 mm biopsy punch (**C**). A rigorous experimental workflow was applied to analyze the mRNA transcriptome of these human ocular samples (**D**).

The RNA-seq experiment described here characterizes several aspects of the human retina mRNA transcriptome. To investigate transcriptional networks specific to retinal neurons, whole corneas were analyzed as a non-neuronal ocular control tissue (Fig. 1A) for comparison to retinal samples (Fig. 1B). Additionally, to more accurately investigate cone and rod-specific cell type-restricted transcriptional regulation, 3 mm and 6 mm samples of the central and peripheral retina were analyzed respectively (Fig. 1C). Finally, this study also analyzes retinal pigment epithelia (RPE)/choroid tissue adjacent to the 3 mm central retina (Fig. 1C). The RPE is a single cell layer that functions as the barrier between the outer retina and the choroid retinal supply blood^20^. Together, the macular retina and adjacent RPE are the primary sites of pathology associated with the complex retinal disorder AMD. RNAs extracted from these tissues were subjected to a rigorous workflow for robust and accurate analysis of mRNA transcriptional networks in clinically relevant ocular cell and tissue types that will be valuable to our research group’s future studies as well as the vision research community (Fig. 1D).

## Methods

### Tissue collection and processing

This study conformed to Institutional Review Board regulations for use of human tissues at James Madison University (JMU). Informed consent from the donor next-of-kin was collected prior to tissue collection. Left and right pairs of whole globe human donor eyes were curated from the National Disease Research Interchange (NDRI; Philadelphia, PA; Fig. 1a; Table 1, Online-only Table 1). Corneas from each eye were collected by making circular cuts along the limbus. Liberated corneal tissue was rinsed in HBBS -Ca, -Mg, blotted dry and immediately flash frozen and ground into a fine powder using a mortar and pestle super cooled with dry ice. Retinas were exposed by making 4 radial cuts down the side of globes from anterior to posterior, laying the dissected eye flat, and removing the vitreous with forceps (Fig. 1b). A central region of the retina was collected using a 3 mm biopsy punch centered on the macula (Integra Miltex, Rietheim-Weilheim, Germany). After peeling off retinal tissue from 3 mm biopsies, the RPE/choroid layer immediately beneath the central retina was separately collected from each specimen herein referred to as RPE/choroid. Rod-rich retinas were similarly collected from regions of the peripheral retina using a 6 mm biopsy punch (Fig. 1c). Tissues were briefly rinsed in cold HBSS -Ca, -Mg and transferred to 1.5 mL tubes containing RLT + lysis buffer (Qiagen; AllPrep kit) supplemented with 2-Mercaptoethanol (Sigma) and vortexed vigorously to dissociate and lyse the tissue. Ground corneas were similarly transferred to RLT+/BME lysis buffer solution and vortexed. Samples were stored in lysis buffer at −80 °C. Similar tissues from left and right eyes from each donor were pooled into single samples. Whole globes and dissected eyes were imaged using a Leica M80 high performance stereomicroscope equipped with an IC80HD camera. Tissues were collected from donors between the ages of 68–95 years and processed within 50 hours of donor death (Online-only Table 1).

**Table 1:** RNA-seq samples, read metrics, and public SRA accessions.

Sample	Tissue	Read Length (bp)	Million read-pairs	% alignmed	NCBI SRA Data Accession
Human Donor Eye 4	Retina-04	2 × 150	57.2	80.40%	SRR10156244
Human Donor Eye 6	Retina-06	2 × 150	49.4	84.10%	SRR10156243
Human Donor Eye 8	Retina-08	2 × 150	54.2	88.20%	SRR10156232
Human Donor Eye 10	Retina-10	2 × 150	59.2	86.20%	SRR10156221
Human Donor Eye 11	Retina-11	2 × 150	61.2	82.40%	SRR10156213
Human Donor Eye 12	Retina-12	2 × 150	56.6	82.50%	SRR10156212
Human Donor Eye 13	Retina-13	2 × 150	55.2	82.00%	SRR10156211
Human Donor Eye 14	Retina-14	2 × 150	55.2	88.80%	SRR10156210
Human Donor Eye 16	Retina-16	2 × 150	55.6	86.60%	SRR10156209
Human Donor Eye 17	Retina-17	2 × 150	60	81.60%	SRR10156208
Human Donor Eye 18	Retina-18	2 × 150	49.8	85.70%	SRR10156242
Human Donor Eye 19	Retina-19	2 × 150	55.2	90.00%	SRR10156241
Human Donor Eye 4	Macula-04	2 × 150	56.8	86.10%	SRR10156240
Human Donor Eye 6	Macula-06	2 × 150	58.8	88.40%	SRR10156239
Human Donor Eye 8	Macula-08	2 × 150	58.8	85.90%	SRR10156238
Human Donor Eye 10	Macula-10	2 × 150	64.2	87.30%	SRR10156237
Human Donor Eye 11	Macula-11	2 × 150	57.8	88.90%	SRR10156236
Human Donor Eye 12	Macula-12	2 × 150	59.8	87.50%	SRR10156235
Human Donor Eye 13	Macula-13	2 × 150	60.2	88.10%	SRR10156234
Human Donor Eye 14	Macula-14	2 × 150	59.2	87.30%	SRR10156233
Human Donor Eye 16	Macula-16	2 × 150	52.6	87.60%	SRR10156231
Human Donor Eye 17	Macula-17	2 × 150	60.2	87.50%	SRR10156230
Human Donor Eye 18	Macula-18	2 × 150	59.4	86.30%	SRR10156229
Human Donor Eye 19	Macula-19	2 × 150	56.8	88.60%	SRR10156228
Human Donor Eye 7	MaculaRPE-07	2 × 150	46	91.10%	SRR10156219
Human Donor Eye 9	MaculaRPE-09	2 × 150	60.8	88.30%	SRR10156218
Human Donor Eye 13	MaculaRPE-13	2 × 150	58.4	86.80%	SRR10156217
Human Donor Eye 15	MaculaRPE-15	2 × 150	68.6	83.40%	SRR10156216
Human Donor Eye 16	MaculaRPE-16	2 × 150	56.4	73.90%	SRR10156215
Human Donor Eye 18	MaculaRPE-18	2 × 150	56	90.10%	SRR10156214
Human Donor Eye 1	Cornea-01	2 × 150	51	87.80%	SRR10156227
Human Donor Eye 2	Cornea-02	2 × 150	63	83.70%	SRR10156226
Human Donor Eye 3	Cornea-03	2 × 150	62.8	87.50%	SRR10156225
Human Donor Eye 7	Cornea-07	2 × 150	51.8	87.30%	SRR10156224
Human Donor Eye 10	Cornea-10	2 × 150	53.8	86.80%	SRR10156223
Human Donor Eye 15	Cornea-15	2 × 150	63	85.10%	SRR10156222
Human Donor Eye 17	Cornea-17	2 × 150	53.4	89.00%	SRR10156220

### Total RNA isolation

Total RNA was extracted from 37 human ocular tissues using a Qiagen AllPrep Mini Kit (Hilden, Germany) with an on column DNaseI treatment step per the manufacturer’s instructions (Table 1). Isolated RNAs were eluted in nuclease free water, validated for quality and quantity using UV spectrophotometry, and stored at −80 °C. RNAs with a OD260/280 ratio between 1.9 and 2.1 were deemed high quality and used for downstream analysis.

### RNA preparation and sequencing

Total RNA samples were submitted to the Genewiz commercial sequencing facility (South Plainfield, NJ) for Bioanalyzer quality control analysis (Agilent, Santa Clara, CA) and Illumina Next Generation Sequencing. All submitted samples had an RNA integrity number (RIN) > 8. Stranded TruSeq cDNA libraries with poly dT enrichment were prepared from total RNA from each sample according to the manufacture’s protocol. Libraries for the 37 cDNA samples were sequenced using the Illumina HiSeq sequencing platform yielding 23–34.3 million 150 bp paired end (PE) sequence reads per sample (Table 1). 74 PE FASTQ files received back from Genewiz were analyzed using a customized bioinformatics workflow (Fig. 1d).

### Quality validation, trimming, and read alignment

**Fig. 2:**
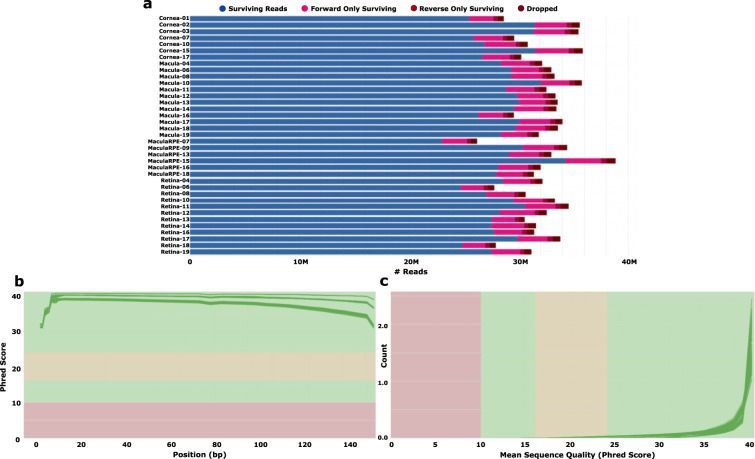
Filtering and quality assessment of raw FASTQ sequencing data. RNA-seq FASTQ files representing the 37 PE samples used in this analysis were filtered and trimmed using Trimmomatic software (**a**). Following trimming and filtering, each of the 74 FASTQ files were assessed for average per base (**b**) and per sequence (**c**) quality as measured by Phred score.

**Fig. 3:**
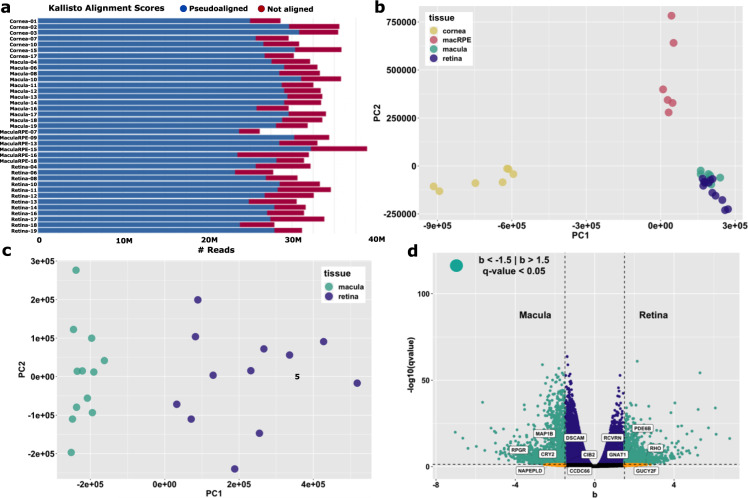
Quality assessment of read alignment and sample variance. The majority of high quality sequence reads successfully aligned to the human hg38 reference transcriptome using the Kallisto pseudoaligner (**a**). Kallisto transcript quantification of each sample was used for two dimensional principal component analysis (PCA) of sample variance with principle component 1 (PC1) and principle component 2 (PC2) accounting for the majority of each sample’s variance. PCA was used to visualize variance between the 4 distinct sample groups as well as similarity within sample replicates for all 37 samples (**b**). PCA was separately applied to 24 tightly clustered central retina and peripheral retina samples (**c**). Differentially expressed transcripts between central and peripheral retina samples plotted based on q-value [-log10(qval)] and fold change [b] demonstrates *RHO*, *PDE6B*, *RCVRN, GUCY2F*, and *GNAT1* are preferentially expressed in the peripheral retina whereas *CIB2*, *MAP1B*, *CRY2*, *NAPEPLD*, *DSCAM*, *CCDC66*, and *RPGR* are representative transcripts preferentially expressed in the central retina (**d**).

Between 23–34.3 million PE sequence reads per sample were delivered from Genewiz (Table 1). Trimmomatic software was used to filter and trim minority low quality sequencing reads from the data set^21^ (see Code availability 1). Figure 2a demonstrates that relatively few reads were filtered out of the data set. Quality of sequence reads in the 74 FASTQ files was evaluated using FastQC analysis^22^ (see Code availability 2), including per base (Fig. 2b) and per sequence (Fig. 2c) analysis which plots the Phred quality score distribution at each base and for each averaged full length read respectively for all reads in the data set. Collectively, Fig. 2 demonstrates that all 74 FASTQ sequencing files have an average per base Phred score >28, a conventional threshold denoting high quality NGS base calls. High quality sequence reads were aligned to the human hg38 reference transcriptome using the ultrafast Kallisto pseudoaligner^23^ (see Code availability 3). The percentage of aligned reads ranged from 73.9 to 91.1% (Table 1; Fig. 3a). Aggregate data visualizations for Trimmomatic, FastQC, and Kallisto were generated using MultiQC software^24^ (see Code availability 4).

### Data transformation and downstream analysis

Transcript quantification of each sample was achieved using Kallisto pseudoalignments. Kallisto outputs were fed into the Sleuth statistical model^25^ for determination of differential transcript expression between samples (see Code availability 5). Sleuth is available as an R package and was used to generate a principal component analysis (PCA) plot demonstrating the variance between distinct sample groups as well as similarity within sample replicates for all 37 samples (Fig. 3b). To specifically highlight the utility of this data set for studying PR-specific cell type-restricted transcriptional networks, Sleuth was separately used for PCA of the tightly clustered 24 central and peripheral retina samples (Fig. 3c). This PCA plot demonstrates distinct clustering amongst 3 mm central retina and 6 mm peripheral retina samples respectively. Kallisto transcript quantification combined with Sleuth statistical analysis was further used to plot all differentially expressed transcripts between central and peripheral retina samples. This analysis demonstrates differential expression of rod-specific transcripts including *RHO*, *PDE6B*, *RCVRN, GUCY2F*, and *GNAT1*. These findings are similar to those observed in transcript analysis of the primate *Macaca fascicularis* central and peripheral retina supporting the practicality of our data set for studying PR cell type-restricted transcription^26^ (Fig. 3d). Curiously, cone-specific transcripts upregulated the central retina of *M. fascicularis* such as *OPN1SW*, *OPN1MW*, *GNAT2*, *ARR3*, and *PDE6H* were not differentially expressed in our study. Transcripts more abundantly expressed in human central retina involved in photoreceptor function include *CIB2*, *MAP1B*, *CRY2*, *NAPEPLD*, *DSCAM*, *CCDC66*, and *RPGR* (Fig. 3c). Discrepancies between human and non-human primate transcript expression patterns in the central retina are one of several areas of current investigation using this data set. Collectively, Fig. 3 demonstrates that the sampling strategy used in our study was effective for comparing differential transcript expression in clinically relevant ocular cell and tissue types.

## Data Records

Raw FASTQ files for the RNA-seq libraries were deposited to the NCBI Sequence Read Archive (SRA), and have been assigned the SRA study accession SRP222833 (Table 1)^27^. Additionally, processed Kallisto output data files for trimmed and pseudoaligned sequence reads are accessible from the Figshare repository for each of the 37 samples analyzed in our study^28^. Kallisto produces three output files per sample:

1. An abundances.h5 HDF5 binary file containing run info, abundance estimates, bootstrap estimates, and transcript length information length. This file can be read in by the sleuth statistical analysis program.
2. An abundances.tsv plaintext file of the abundance estimates. Use the–plaintext mode to output plaintext abundance estimates. Alternatively, kallisto h5dump can be used to output an HDF5 file to plaintext. The first line contains a header for each column, including estimated counts, TPM, effective length.
3. A run_info.json file containing information about the run.

## Technical Validation

### Quality control-RNA integrity

Quality of total RNA fractions was assessed using an Agilent Bioanalyzer to calculate a RNA Integrity Number (RIN). The RIN algorithm determines the RNA quality of the samples with the highest quality having a score of 10. Conventional to NGS analysis, only RNA samples with a RIN > 8 were used for sequencing analysis.

### RNA-Seq raw data quality and filtering

Trimmomatic was used to filter and trim minority low quality sequencing reads and bases from downstream analysis (Fig. 2a). FastQC per base and per sequence quality analysis demonstrates mean Phred quality scores are well within the acceptable range for downstream analysis (Fig. 2b,c). Between 23 and 34.3 million reads were mapped to the human hg38 transcriptome assembly (Fig. 3a; Table 1). PCA biplot analysis confirmed the similarity between biological replicates and variability between tissue samples respectively (Fig. 3b,c).

## Usage Notes

The bioinformatics pipeline applied to our data set outlined in Fig. 1d was achieved using a collection of freely available, open access tools. These analyses however, are interchangeable with many other currently available tools for achieving different experimental outcomes. Our raw FASTQ data can be aligned to any available human reference genome or transcriptome, including the most recent 2013 hg38 reference assemblies using a variety of freely available aligners. In this study we used Kallisto, an alignment-free transcriptome pseudoaligner^23^, with the specific interest in expression quantification of previously characterized mRNA isoforms. An alignment-free pipeline significantly reduces the time of analysis as well as required computing power and file storage, which may be beneficial for some users. Other very fast alignment-free programs such as Sailfish^29^ and Salmon^30^ can be used to achieve similar expression quantification analysis with these data. Alignment-free programs however, are not suitable for novel isoform analysis. More traditional alignment-based RNA-seq pipelines such as the ‘new tuxedo’ suite can be used to analyze these data for novel isoforms^31^. Here our differential gene expression analysis was carried out using the Sleuth software^25^, however other publicly available packages such as edgeR^32^ or StringTie^31^ may also be used for similar analysis. Importantly, QC data presented in Fig. 2 and Fig. 3 demonstrate the high quality of sequencing reads and precision of sampling respectively making this data set compatible with alignment tools currently available as well as new alignment tools that may become available in the future.

Our data set will be useful for a variety of studies investigating cell type and tissue-specific patterns of gene expression in the human retina, RPE/choroid, and cornea as well as diseases that affect these tissues. In particular, this work will build on existing genomic data sets investigating the human retina and AMD in particular. Fritsche and colleague’s landmark GWAS identified 52 SNPs at 32 loci constituting the majority of AMD heritability^33^. A recent transcriptome study integrating AMD GWAS data with RNA-seq data from 453 postmortem human retina samples identified three novel candidate AMD-associated genes^34^. Notably, Ratnapriya and colleagues collected their RNA-seq data from whole retina. In contrast, the study presented here focuses more specifically on primary sites of AMD pathology thereby providing a unique data set to the vision research community.

Several considerations must be taken into account when using these data for downstream analysis. First, RNAs were extracted from retinal tissue enriched in rod and cone photoreceptors, RPE/choroid, and whole cornea without any further enrichment for cell type. Therefore, resulting downstream analysis will be representative of heterogeneous mixtures of differing cell types within these tissues. In particular, 3 mm biopsies of the central retina sample a subsection of the 5.5 mm diameter macula encompassing the fovea, parafovea and portions of the perifovea. Though this cone-rich region contains some rod PRs, our data demonstrates that rod-specific transcripts are present at much lower counts compared to adjacent peripheral retina (Fig. 3d). These data suggest that cones are the predominant PR cell type in these samples, though notably, several canonical cone-specific genes (*OPN1SW*, *OPN1MW*, *GNAT2*, *ARR3*, *PDE6H)* were not differentially expressed in our study. Second, cDNA libraries were prepared using a poly dT primer, thus the data set is representative of only polyadenylated transcripts and does not represent many non-coding RNA or other non-polyadenylated cellular transcripts. Additionally, usage of poly dT priming introduces a bias towards overrepresentation of the 3′ end of transcripts, particularly in the case of relatively large transcripts. Finally, the quantity of sequenced and mapped reads per sample in this study (Table 1; Fig. 3a) is sufficient for robust differential transcript/gene expression analysis, however, is below the conventional threshold for thorough differential isoform analysis^35^. Taking these considerations into account, these data will be a useful resource for the vision research community for robust and accurate analysis of polyadenylated transcriptional networks in clinically relevant ocular cell and tissue types.

## Data Availability

The following open access software and versions were used for quality control and data analysis as described in the main text: Trimmomatic, version 0.36 was used to filter and trim low quality reads and bases from FASTQ sequencing data files: http://www.usadellab.org/cms/?page=trimmomatic FastQC, version 0.11.5 was used for quality analysis of raw FASTQ sequencing data: http://www.bioinformatics.babraham.ac.uk/projects/fastqc/ Kallisto, version 0.42.3 was used to index and psedudoalign sequencing reads to the human hg38 transcriptome as well as to quantify transcripts in each samples: https://pachterlab.github.io/kallisto/ MultiQC, was used to aggregate and visualize FastQC, Trimmomatic, and Kallisto data outputs: https://multiqc.info/ Sleuth, was used to analyze transcript abundances quantified by Kallisto as well as to calculate and summarize differential transcript expression: https://pachterlab.github.io/sleuth/about All code and walkthroughs used for quality assessment and data analysis in this study is available at: https://github.com/enkera/Schumacker2019_Sci_Data

## Online-only Table

**Online-only Table 1:** Associated RNA-seq sample metadata.

Source Name (mandatory)	Characteristics[death:tissue delivery time]	Characteristics[organism]	Characteristics[organism part]	Total RNA isolation	RNA preparation and sequencing	Independent Variable[donor age]	Independent Variable[donor sex]	Sample Name (mandatory)
*Your starting materials. Maybe physical objects (e.g. mice, chemicals), digital objects (e.g. published articles). Add a name/identifier for the source that the sample came from. Multiple samples may come from a single source*. *NO SPECIAL CHARACTERS: ‘% + &;()[]{}àáâãäç etc*	*What would future users of your data need to know about your sources and samples? Please change VARIABLE_NAME to something appropriate (e.g.: developmental stage, cell line, strain). Add columns for all Characteristics you think are important for understanding and reusing your data*.	*This Characteristic is recommended for biological & medical studies. Delete this column if not applicable*.	*This Characteristic recommended for biological & medical studies. Delete this column if not applicable*.	*What did you do to your Sources to get your Samples? Each method should match a sub-heading in the Methods section of your manuscript*.	*Duplicate Method column as many times as needed to describe how you got your Samples (or delete this column if not applicable)*.	*Any independent variables that were part of your experimental set-up. Please change VARIABLE_NAME to something appropriate (e.g.: date, brain region, staining technique). Add columns as needed*.	*Any independent variables that were part of your experimental set-up. Please change VARIABLE_NAME to something appropriate (e.g.: date, brain region, staining technique). Add columns as needed*.	*Give a unique identifier to each sample that you used to create your data*. *NO SPECIAL CHARACTERS: ‘% + &;()[]{}àáâãäç etc*
Human Donor Eye 4	43 hours	Homo sapiens	peripheral retina	Qiagen AllPrep Mini Kit	Illumina stranded TruSeq cDNA libraries with poly dT enrichment; Illumina HiSeq sequencer	82 years	male	retina_4
Human Donor Eye 6	34 hours	Homo sapiens	peripheral retina	Qiagen AllPrep Mini Kit	Illumina stranded TruSeq cDNA libraries with poly dT enrichment; Illumina HiSeq sequencer	79 years	female	retina_6
Human Donor Eye 8	46 hours	Homo sapiens	peripheral retina	Qiagen AllPrep Mini Kit	Illumina stranded TruSeq cDNA libraries with poly dT enrichment; Illumina HiSeq sequencer	78 years	male	retina_8
Human Donor Eye 10	45 hours	Homo sapiens	peripheral retina	Qiagen AllPrep Mini Kit	Illumina stranded TruSeq cDNA libraries with poly dT enrichment; Illumina HiSeq sequencer	89 years	male	retina_10
Human Donor Eye 11	50 hours	Homo sapiens	peripheral retina	Qiagen AllPrep Mini Kit	Illumina stranded TruSeq cDNA libraries with poly dT enrichment; Illumina HiSeq sequencer	68 years	male	retina_11
Human Donor Eye 12	26 hours	Homo sapiens	peripheral retina	Qiagen AllPrep Mini Kit	Illumina stranded TruSeq cDNA libraries with poly dT enrichment; Illumina HiSeq sequencer	92 years	male	retina_12
Human Donor Eye 13	26 hours	Homo sapiens	peripheral retina	Qiagen AllPrep Mini Kit	Illumina stranded TruSeq cDNA libraries with poly dT enrichment; Illumina HiSeq sequencer	69 years	male	retina_13
Human Donor Eye 14	41 hours	Homo sapiens	peripheral retina	Qiagen AllPrep Mini Kit	Illumina stranded TruSeq cDNA libraries with poly dT enrichment; Illumina HiSeq sequencer	75 years	male	retina_14
Human Donor Eye 16	35 hours	Homo sapiens	peripheral retina	Qiagen AllPrep Mini Kit	Illumina stranded TruSeq cDNA libraries with poly dT enrichment; Illumina HiSeq sequencer	73 years	male	retina_16
Human Donor Eye 17	35 hours	Homo sapiens	peripheral retina	Qiagen AllPrep Mini Kit	Illumina stranded TruSeq cDNA libraries with poly dT enrichment; Illumina HiSeq sequencer	95 years	male	retina_17
Human Donor Eye 18	24 hours	Homo sapiens	peripheral retina	Qiagen AllPrep Mini Kit	Illumina stranded TruSeq cDNA libraries with poly dT enrichment; Illumina HiSeq sequencer	93 years	male	retina_18
Human Donor Eye 19	43 hours	Homo sapiens	peripheral retina	Qiagen AllPrep Mini Kit	Illumina stranded TruSeq cDNA libraries with poly dT enrichment; Illumina HiSeq sequencer	68 years	male	retina_19
Human Donor Eye 4	43 hours	Homo sapiens	macular retina	Qiagen AllPrep Mini Kit	Illumina stranded TruSeq cDNA libraries with poly dT enrichment; Illumina HiSeq sequencer	82 years	male	macula_4
Human Donor Eye 6	34 hours	Homo sapiens	macular retina	Qiagen AllPrep Mini Kit	Illumina stranded TruSeq cDNA libraries with poly dT enrichment; Illumina HiSeq sequencer	79 years	female	macula_6
Human Donor Eye 8	46 hours	Homo sapiens	macular retina	Qiagen AllPrep Mini Kit	Illumina stranded TruSeq cDNA libraries with poly dT enrichment; Illumina HiSeq sequencer	78 years	male	macula_8
Human Donor Eye 10	45 hours	Homo sapiens	macular retina	Qiagen AllPrep Mini Kit	Illumina stranded TruSeq cDNA libraries with poly dT enrichment; Illumina HiSeq sequencer	89 years	male	macula_10
Human Donor Eye 11	50 hours	Homo sapiens	macular retina	Qiagen AllPrep Mini Kit	Illumina stranded TruSeq cDNA libraries with poly dT enrichment; Illumina HiSeq sequencer	68 years	male	macula_11
Human Donor Eye 12	26 hours	Homo sapiens	macular retina	Qiagen AllPrep Mini Kit	Illumina stranded TruSeq cDNA libraries with poly dT enrichment; Illumina HiSeq sequencer	92 years	male	macula_12
Human Donor Eye 13	26 hours	Homo sapiens	macular retina	Qiagen AllPrep Mini Kit	Illumina stranded TruSeq cDNA libraries with poly dT enrichment; Illumina HiSeq sequencer	69 years	male	macula_13
Human Donor Eye 14	41 hours	Homo sapiens	macular retina	Qiagen AllPrep Mini Kit	Illumina stranded TruSeq cDNA libraries with poly dT enrichment; Illumina HiSeq sequencer	75 years	male	macula_14
Human Donor Eye 16	35 hours	Homo sapiens	macular retina	Qiagen AllPrep Mini Kit	Illumina stranded TruSeq cDNA libraries with poly dT enrichment; Illumina HiSeq sequencer	73 years	male	macula_16
Human Donor Eye 17	35 hours	Homo sapiens	macular retina	Qiagen AllPrep Mini Kit	Illumina stranded TruSeq cDNA libraries with poly dT enrichment; Illumina HiSeq sequencer	95 years	male	macula_17
Human Donor Eye 18	24 hours	Homo sapiens	macular retina	Qiagen AllPrep Mini Kit	Illumina stranded TruSeq cDNA libraries with poly dT enrichment; Illumina HiSeq sequencer	93 years	male	macula_18
Human Donor Eye 19	43 hours	Homo sapiens	macular retina	Qiagen AllPrep Mini Kit	Illumina stranded TruSeq cDNA libraries with poly dT enrichment; Illumina HiSeq sequencer	68 years	male	macula_19
Human Donor Eye 7	27 hours	Homo sapiens	macular RPE/choroid	Qiagen AllPrep Mini Kit	Illumina stranded TruSeq cDNA libraries with poly dT enrichment; Illumina HiSeq sequencer	75 years	male	RPE_choroid_7
Human Donor Eye 9	36 hours	Homo sapiens	macular RPE/choroid	Qiagen AllPrep Mini Kit	Illumina stranded TruSeq cDNA libraries with poly dT enrichment; Illumina HiSeq sequencer	82 years	male	RPE_choroid_9
Human Donor Eye 13	26 hours	Homo sapiens	macular RPE/choroid	Qiagen AllPrep Mini Kit	Illumina stranded TruSeq cDNA libraries with poly dT enrichment; Illumina HiSeq sequencer	69 years	male	RPE_choroid_13
Human Donor Eye 15	36 hours	Homo sapiens	macular RPE/choroid	Qiagen AllPrep Mini Kit	Illumina stranded TruSeq cDNA libraries with poly dT enrichment; Illumina HiSeq sequencer	80 years	male	RPE_choroid_15
Human Donor Eye 16	35 hours	Homo sapiens	macular RPE/choroid	Qiagen AllPrep Mini Kit	Illumina stranded TruSeq cDNA libraries with poly dT enrichment; Illumina HiSeq sequencer	73 years	male	RPE_choroid_16
Human Donor Eye 18	24 hours	Homo sapiens	macular RPE/choroid	Qiagen AllPrep Mini Kit	Illumina stranded TruSeq cDNA libraries with poly dT enrichment; Illumina HiSeq sequencer	93 years	male	RPE_choroid_18
Human Donor Eye 1	41 hours	Homo sapiens	whole cornea	Qiagen AllPrep Mini Kit	Illumina stranded TruSeq cDNA libraries with poly dT enrichment; Illumina HiSeq sequencer	70 years	male	cornea_1
Human Donor Eye 2	43 hours	Homo sapiens	whole cornea	Qiagen AllPrep Mini Kit	Illumina stranded TruSeq cDNA libraries with poly dT enrichment; Illumina HiSeq sequencer	76 years	male	cornea_2
Human Donor Eye 3	34 hours	Homo sapiens	whole cornea	Qiagen AllPrep Mini Kit	Illumina stranded TruSeq cDNA libraries with poly dT enrichment; Illumina HiSeq sequencer	71 years	male	cornea_3
Human Donor Eye 7	27 hours	Homo sapiens	whole cornea	Qiagen AllPrep Mini Kit	Illumina stranded TruSeq cDNA libraries with poly dT enrichment; Illumina HiSeq sequencer	75 years	male	cornea_7
Human Donor Eye 10	45 hours	Homo sapiens	whole cornea	Qiagen AllPrep Mini Kit	Illumina stranded TruSeq cDNA libraries with poly dT enrichment; Illumina HiSeq sequencer	89 years	male	cornea_10
Human Donor Eye 15	36 hours	Homo sapiens	whole cornea	Qiagen AllPrep Mini Kit	Illumina stranded TruSeq cDNA libraries with poly dT enrichment; Illumina HiSeq sequencer	80 years	male	cornea_15
Human Donor Eye 17	35 hours	Homo sapiens	whole cornea	Qiagen AllPrep Mini Kit	Illumina stranded TruSeq cDNA libraries with poly dT enrichment; Illumina HiSeq sequencer	95 years	male	cornea_17
